# Solving the Problem of Comparing Whole Bacterial Genomes across Different Sequencing Platforms

**DOI:** 10.1371/journal.pone.0104984

**Published:** 2014-08-11

**Authors:** Rolf S. Kaas, Pimlapas Leekitcharoenphon, Frank M. Aarestrup, Ole Lund

**Affiliations:** 1 National Food Institute, Technical University of Denmark, Lyngby, Denmark; 2 Center for Biological Sequence Analysis, Technical University of Denmark, Lyngby, Denmark; University Medical Center Groningen, Netherlands

## Abstract

Whole genome sequencing (WGS) shows great potential for real-time monitoring and identification of infectious disease outbreaks. However, rapid and reliable comparison of data generated in multiple laboratories and using multiple technologies is essential. So far studies have focused on using one technology because each technology has a systematic bias making integration of data generated from different platforms difficult. We developed two different procedures for identifying variable sites and inferring phylogenies in WGS data across multiple platforms. The methods were evaluated on three bacterial data sets and sequenced on three different platforms (Illumina, 454, Ion Torrent). We show that the methods are able to overcome the systematic biases caused by the sequencers and infer the expected phylogenies. It is concluded that the cause of the success of these new procedures is due to a validation of all informative sites that are included in the analysis. The procedures are available as web tools.

## Introduction

Microbial whole-genome sequencing using bench-top sequencing technologies holds great promises to enhance diagnostic and public health microbiology [1]–[3]. Its great value in describing and improving our understanding of bacterial evolution, outbreaks and transmission events has been shown in a number of recent studies, including *Staphylococcus aureus* [4]–[6], *Vibrio cholera* [7], *Escherichia coli* [8], *Mycobacterium tuberculosis* [9] and surveillance of antimicrobial resistance [10]. All of these studies have however, been done retrospectively, (except [8], which was done prospectively) and conducted using the same technology and performed in a single laboratory.

For rapid detection of out-breaks involving multiple sites or even countries it is essential to enable rapid and reliable comparison of data generated in different laboratories and using different technologies [1]. Enabling comparison between technologies is also important for the future comparison of data generated using novel technologies that are currently under development and comparison to data already generated using current technologies. An important step to enable this is to allow for sequencing platform independent analysis. This is especially relevant for SNP calling where the currently available sequencing platforms all have some type of systematic sequencing bias [11]–[16]. These systematic biases’ today make it virtually impossible to perform reliable phylogenetic studies if the data are generated using different technologies. For research purposes the correct identification of SNP’s might be solved by sequencing using multiple platforms, but for infectious disease out-breaks this will neither be practical or timely feasible. Infectious disease out-breaks are often multistate and rapid comparison and correct clustering is essential.

Common practice in SNP calling is to use a closely related reference genome, often a reference genome that has been sequenced and finished with respect to the study in question. While this approach is feasible for research purposes it is not practical in an out-break investigation.

In this study we developed two novel procedures for identifying variations in whole genome sequencing reads and conducting phylogenetic analysis of isolates. The procedures were evaluated on an available data-set where three different platforms had been used to sequence the same 12 *Salmonella* Montevideo isolates, as well as sequencing of selected *Salmonella* Typhimurium and *Staphylococcus aureus* isolates using Illumina and Life Technologies.

The novel procedures have been made available as web tools at the following addresses:

Nucleotide Difference (ND) method: http://cge.cbs.dtu.dk/services/NDtree/.

Novel SNP procedure: http://cge.cbs.dtu.dk/services/CSIPhylogeny/.

## Materials and Methods

### Datasets

Three different datasets were used for evaluation in the present study, comprising selected *Salmonella* Montevideo [17], *Staphylococcus aureus* CC398 [5], and *Salmonella* Typhimurium DT104 [18] from previous studies.

For *S*. Montevideo 12 closely related outbreak strains where sequenced once by US Food and Drug Administration using Roche Genome sequencer FLX system, Illumina MiSeq and Life Technologies Ion Torrent and made publicly available (Table S1), although only the MiSeq data was used in the original study [16]. The raw data were downloaded from the Sequence Read Archive (SRA). For *Staphylococcus aureus* CC398, the completely sequenced and annotated strain SO385 (AM990992.1) as well as four additional strains were selected from a previously published study [5] and sequenced twice using both MiSeq and Ion Torrent. HiSeq was used in the original study for sequencing. All the strains except for the reference strain were chosen from the same clade, named IIa1i in the original study. The strains are not epidemiologically related but have all been isolated from Danish Pigs and are shown to be closely related in the original study. For S. Typhimurium DT104 the reference strain NCTC 13348 (HF937208.1) and an additional three isolates from the same outbreak [18] were sequenced twice on both MiSeq and Ion Torrent.

Genomic DNA (gDNA) was purified from the isolates using the Easy-DNA extraction kit (Invitrogen) and DNA concentrations determined using the Qubit dsDNA BR Assay Kit (Invitrogen). The isolates were sequenced twice on the MiSeq platform (Illumina) and Ion Torrent PGM (Life Technologies).

For Ion Torrent the isolates were sequenced following the manufacturer’s protocols for 200 bp gDNA fragment library preparation (Ion Xpress Plus gDNA and Amplicon Library 96 Preparation), template preparation (Ion OneTouch System), and sequencing (Ion PGM 200 Sequencing kit) using the 316 chip. For MiSeq the isolates chromosomal DNA of the isolates was used to create genomic libraries using the Nextera XT DNA sample preparation kit (Illumina, cat. No. FC-131-1024) and sequenced using v2, 2×250 bp chemistry on the Illumina MiSeq platform (Illumina, Inc., San Diego, CA).

### Data analysis

The raw data was trimmed and cleaned for adapters using AdapterRemoval v. 1.1 (https://code.google.com/p/adapterremoval/) before any analysis was done.

The data were analyzed using an available and published pipeline for SNP-calling and creation of phylogenetic trees [19], a recently developed method based on nucleotide differences [18], as well as a novel procedure for SNP-calling developed in this study. All three methods requires a reference sequence, these has been listed in Table 1. All the references applied in this study are available as complete assemblies from GenBank.

**Table 1:** Reference Genomes.

Ref. genome	Distance	Size (bp)	Accession No.
*S. aureus* CC398	close	2,872,582	AM990992.1
*S. aureus* ST228	distant	2,759,835	NC_020533.1
*S.* DT104	close	4,933,631	HF937208.1
*S.* Schwarzengrund	distant	4,709,075	NC_011094.1

#### Nucleotide Difference (ND) procedure (Novel)

A previously published procedure [18] was used. In Brief, each read were mapped to the reference genome. A base was called if Z = (X−Y)/sqrt(X+Y) was greater than 1.96 corresponding to a p-value of 0.05. Here X is the number of reads X having the most common nucleotide at that position, and Y the number of reads supporting other nucleotides. It was further required that X>10*Y. The number of nucleotide differences in positions called in all sequences was counted, and a matrix with these counts was given as input to an UPGMA algorithm implemented in the neighbor program v. 3.69 (http://evolution.genetics.washington.edu/phylip.html) in order to construct the tree.

#### SNP analysis (Novel)

Reads were mapped to reference sequences using BWA v. 0.7.2 [20]. The depth at each mapped position was calculated using genomeCoverageBed, which is part of BEDTools v. 2.16.2 [21]. Single nucleotide polymorphisms (SNPs) were called using mpileup part of SAMTools v. 0.1.18 [22]. SNPs were filtered out if the depth at the SNP position was not at least 10x or at least 10% of the average depth for the particular genome mapping. The reason for applying a relative depth filter is to set different thresholds for sequencing runs that yield very different amounts of output data (total bases sequenced). SNPs were filtered out if the mapping quality was below 25 or the SNP quality was below 30. The quality scores were calculated by BWA and SAMTools, respectively. The scores are phred-based but can be converted to probabilistic scores, with the formula 10∧(−Q/10), where Q is the respective quality score. The probabilistic scores will represent the probability of a wrong alignment or an incorrect SNP call, respectively. In each mapping, SNPs were filtered out if they were called within the vicinity of 10 bp of another SNP (pruning). A Z-score was calculated for each SNP as described above for NDtree.

The depth requirements ensure that all positions considered are covered by a minimum amount of reads. The SNP quality and the Z-score requirements ensures that all positions considered are also called with significant confidence with respect to the bases called at each position.

All genome mappings were then compared and all positions where SNPs was called in at least one mapping were validated in all mappings. The validation includes both the depth check and the Z-score check as for the SNP filtering. Any position that fails validation is ignored in all mappings.

Maximum Likelihood trees were created using FastTree [23].

#### snpTree

Analysis was done using the method described by Leekitcharoenphon et al. [19]. The primary difference between the snpTree method and the novel SNP analysis is in the filtering and validation of the SNP positions. Briefly, the snpTree method calls SNPs using BWA [20], then the default behavior is to filter out SNPs with a depth less than 10 and SNPs found within 10 bps of each other (pruning). An alignment of the SNPs are then created by concatenating the SNPs. Positions where no SNPs are found or where SNPs has been ignored are assumed to be identical to the base in the reference sequence. A maximum likelihood tree is created from the alignment.

## Results

**Figure 1:**
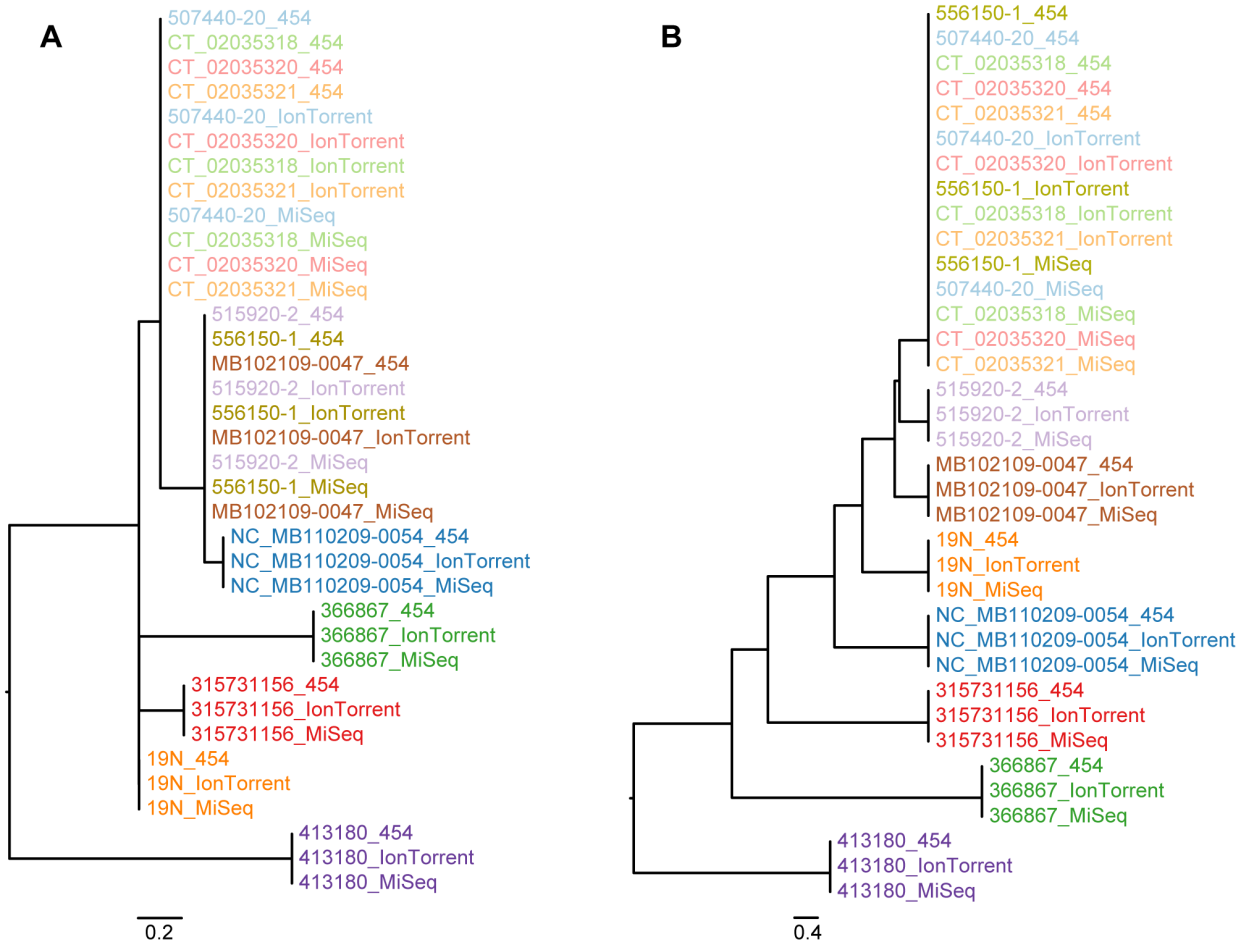
*Salmonella* Montevideo phylogeny (complete dataset). Labels are colored according to isolate. The sequencing platforms applied are appended to the end of each label. (**A**) Phylogeny inferred with novel SNP procedure; (**B**) Phylogeny inferred with the Nucleotide Difference (ND) method.

**Figure 2:**
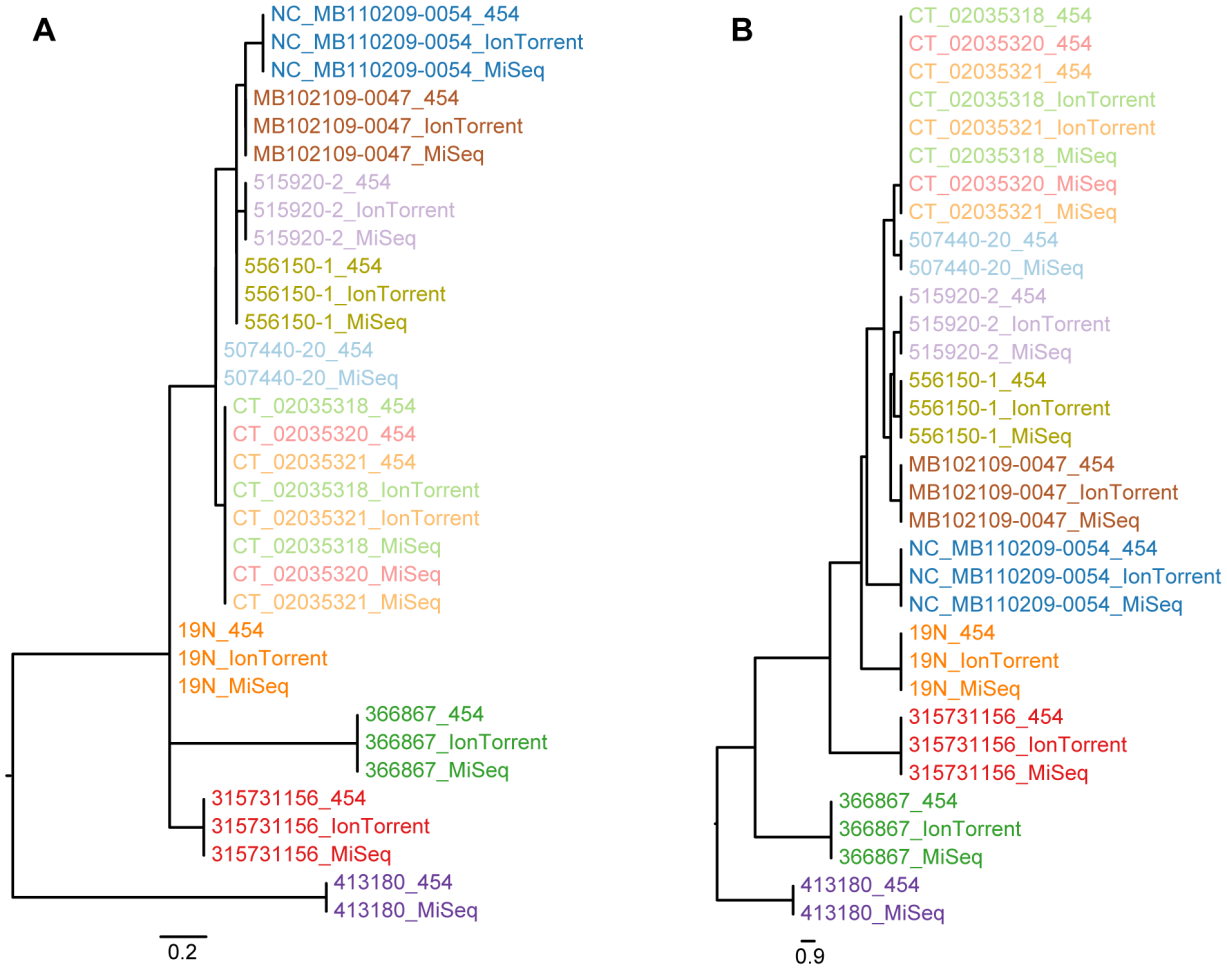
*Salmonella* Montevideo phylogeny (low quality sequences removed). Labels are colored according to isolate. The sequencing platforms applied are appended to the end of each label. (**A**) Phylogeny inferred with novel SNP procedure; (**B**) Phylogeny inferred with the Nucleotide Difference (ND) method.

**Figure 3:**
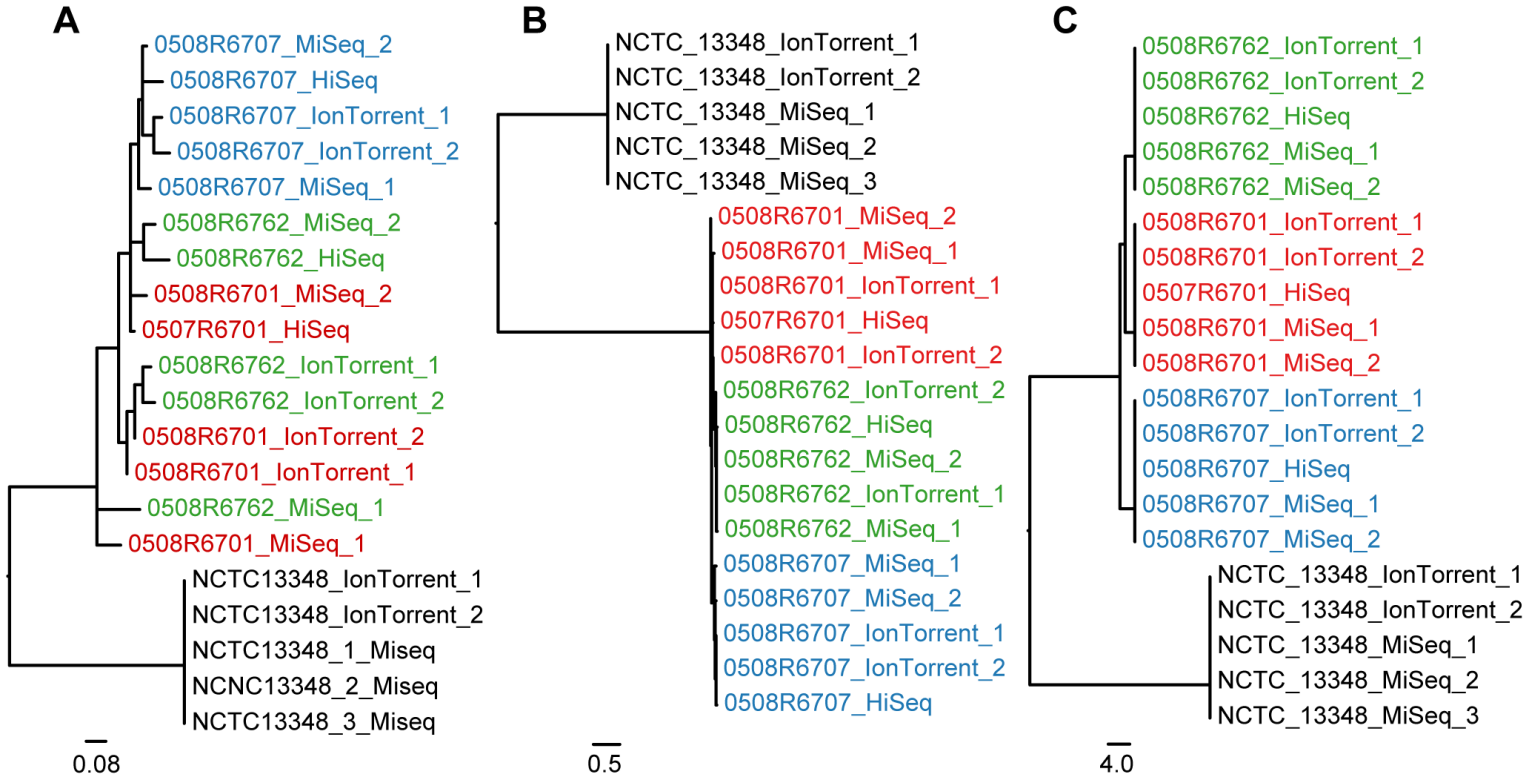
*Salmonella* DT104 phylogeny. Labels are colored according to isolate. The sequencing platforms applied are appended to the end of each label. If repetitive sequencing has been performed then the label has also been appended either “1” or “2”. (**A**) Phylogeny inferred with snpTree; (**B**) Phylogeny inferred with the novel SNP procedure; (**C**) Phylogeny inferred with the Nucleotide Difference (ND) method.

**Figure 4:**
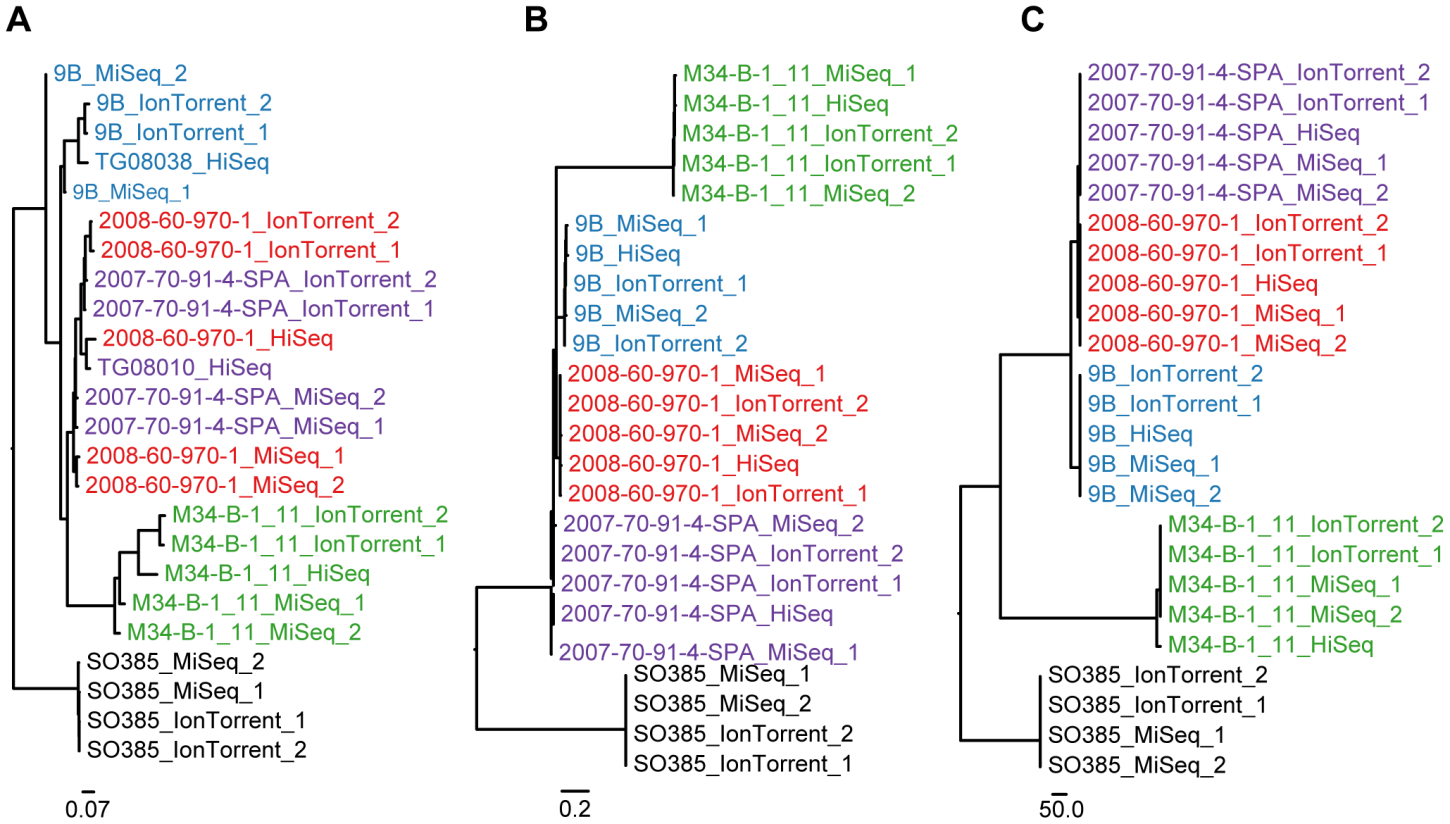
*Staphylococcus aureus* phylogeny. Labels are colored according to isolate. The sequencing platforms applied are appended to the end of each label. If repetitive sequencing has been performed then the label has also been appended either “1” or “2”. (**A**) Phylogeny inferred with snpTree; (**B**) Phylogeny inferred with the novel SNP procedure; (**C**) Phylogeny inferred with the Nucleotide Difference (ND) method.

A comparison of the three different methods is given in Table 2 and Figure 1–4 for the different datasets. The original procedure (snpTree) was un-able to cluster the same isolates correctly across the different technologies whereas both of the novel methods gave improved results.

**Table 2:** Comparison of the novel SNP procedure, the Nucleotide Difference (ND) method and snpTree.

Method	Percent of reference genome covered				
	*S.* Montevideo	*S.* DT104		*S. aureus*	
	*Distant ref.*	*Distant ref.*	*Close ref.*	*Distant ref.*	*Close ref.*
snpTree	100.00	100.00	100.00	100.00	100.00
novel SNP	81.40	92.48	99.42	93.05	99.40
ND	34.48	88.60	95.68	63.44	88.00
	**Informative sites**				
snpTree	22068	26691	79	20324	699
novel SNP	18 (36)	49	66	107	252
ND	19 (33)	54	66	126	602
	**Average distance within clusters**				
snpTree	6353.0	8024.0	8.1	4271.0	69.0
novel SNP	0 (0)	1.0	1.5	1.0	2.0
ND	0 (0)	0.0	0.0	2.0	3.6

snpTree does not ignore any positions and is potentially able to consider 100% of the genome. The novel SNP procedure considers between 81.40% and 99.42%. The ND method is more conservative and considers between 34.48% and 95.68%. The snpTree method was expected to have issues with the references that were distantly related as also mentioned by the authors of this method. This is also illustrated in Table 2 by the large amount of informative SNPs that the method finds compared to the other methods, when the references are distantly related to the analyzed isolates. A plot of the number of positions that each isolate causes to be ignored in the Montevideo analysis (see Figure S1) shows very clearly that three isolates causes more than half of the ignored positions. The three isolates were deemed of low quality, removed from the analysis, and the methods were rerun. The numbers from the rerun is presented in parentheses in Table 2.

### Salmonella Montevideo

Each of the three methods was applied to just the MiSeq data and compared to the SNP tree published by Allard et al. [17] (Figures S2, S3, and S4). The novel SNP procedure infers a phylogeny that agrees with the published one. The ND procedure infers a tree that almost agrees with the published one, except that the “clinical clade” is reversed with respect to the most recent common forefathers. The snpTree method infers a phylogeny that is very different from the published one and will therefore not be discussed here (Figure S2).

Figure 1A and 1B presents the phylogeny that was inferred by applying the entire Montevideo dataset to the novel SNP procedure and the ND method, respectively. Compared to the MiSeq only phylogeny it is observed that the phylogeny has lost a lot of resolution, but in general keeps the same topology, as the respective phylogenies inferred with the MiSeq data alone.

Figure 2A and 2B presents the phylogeny that was inferred by leaving out the three isolates with low quality sequence data. The topology generally remains the same but much more resolution is provided in these phylogenies. The increased resolution is explained by the increase of informative sites, which are doubled with the novel SNP procedure and also close to doubled with the ND method.

### Salmonella Typhimurium DT104

snpTree seems to have problems differentiating properly between the sequence of the isolates that are closely related (Figure 3A), even with a closely related reference. Applying a distantly related reference a clear clustering of platforms and not isolates is seen (Figure S5). The ND method and the novel SNP procedure both cluster the isolates correctly (Figures 3C and 3B). The two methods create two identical phylogenies regardless of the distance to the reference used (see Figures S6 and S7 for phylogenies inferred with a distant reference). The novel SNP method finds between 1 and 1.5 SNPs on average between identical isolates. The ND method finds none.

### Staphylococcus aureus CC398

Even with a close reference snpTree is not able to cluster the isolates 2008-60-970-1 and 2007-70-91-4-SPA correctly. These two isolates are clearly clustered according to sequencing platform and not their true relationship (Figure 4A), this clustering into sequencing platform is very clear if the distant reference is applied (Figure S8). The ND method and the novel SNP procedure both cluster the isolates correctly (Figure 4B and 4C). The ND method again infers phylogenies that are identical regardless of the distance to the reference. The novel SNP procedure infers phylogenies that are almost identical. The difference is with regard to the exact location of the node that leads to the M34-B-1_11 cluster. It is interesting that the phylogenies inferred with close references are so identical to the ones inferred by the distant references, even though the amount of informative sites increases so dramatically (see Table 2). Phylogenies inferred with a distant reference are presented in Figures S9 and S10.

## Discussion

Infectious disease outbreaks often involve isolation of the causative agent in multiple laboratories within a country or even from multiple countries. Early detection of out-breaks thus, often requires rapid comparison of data from different laboratories. Next-generation sequencing shows great promises to improve the routine characterization of infectious disease agents in microbial laboratories and sequencing data are attractive because they both provide high resolution as well as a standardized data format (the DNA sequence) that may be exchanged and compared between laboratories and over time. A number of different sequencing technologies are however, available and more are expected to become available in the future. Thus, the problem with systematic biases in SNP calling between platforms may be a problem especially when, as often the cause in outbreak detection, it is necessary to identify clusters within highly similar strains.

To our knowledge we have provided the first evaluation of phylogenetic analysis done on bacterial isolates sequenced more than once and across platforms. The main reason for the success of the presented methods is in the validation of all the sites, which are part of the phylogenetic analysis. If a position is informative then that position must be called with confidence in all strains, which are part of the analysis. This validation will be very sensitive to low quality sequences. A single low quality sequencing run can cause a lot of informative sites to be ignored. However this would not cause wrong phylogenies but most likely low resolution phylogenies and the analysis, will as presented in this study clearly show which sequences to rerun or leave out and another phylogenetic analysis can quickly be done without the low quality sequences, since the mapping of read data to the reference and most of the calculations has already been done.

The presented procedures may not be perfect in identifying all single SNPs and variable sites, but for routine epidemiological typing of infectious disease agents this is less important than the correct clustering. Further evaluation also under real-time situations as done by Joensen et al. [24] are warranted, but if validated the current or modified procedures may greatly enhance our ability to compare data produced using different sequencing technologies and also provide further comparability with future technologies. The same or similar procedures might also be useful for future large-scale phylogenetic studies on human and other eukaryotic genomes.

## Supporting Information

Figure S1 **Ignored genome positions in novel SNP procedure (** ***Salmonella*** **Montevideo dataset).** Each cluster of three columns represents the amount of genome locations that are ignored due to the addition of the specific data. Black represents MiSeq data, grey represents Ion Torrent data, and light grey represents 454 data.
(PDF)

Figure S2 ***Salmonella*** **Montevideo phylogeny inferred by snpTree (MiSeq data only).** The colors of the labels in the figure correspond to the colors used in the main figures.
(PDF)

Figure S3 ***Salmonella*** **Montevideo phylogeny inferred by the novel SNP procedure (MiSeq data only).** The colors of the labels in the figure correspond to the colors used in the main figures.
(PDF)

Figure S4 ***Salmonella*** **Montevideo phylogeny inferred by the Nucleotide Difference method (MiSeq data only).** The colors of the labels in the figure correspond to the colors used in the main figures.
(PDF)

Figure S5 ***Salmonella*** **DT104 phylogeny inferred with snpTree (distant reference).** Colors have been omitted from this figure. The sequencing platforms applied are appended to the end of each label. If repetitive sequencing has been performed then the label has also been appended either “1” or “2”.
(PDF)

Figure S6 ***Salmonella*** **DT104 phylogeny inferred with the novel SNP procedure (distant reference).** Labels are colored according to isolate. The sequencing platforms applied are appended to the end of each label. If repetitive sequencing has been performed then the label has also been appended either “1” or “2”.
(PDF)

Figure S7 ***Salmonella*** **DT104 phylogeny inferred with the Nucleotide Difference method (distant reference).** Labels are colored according to isolate. The sequencing platforms applied are appended to the end of each label. If repetitive sequencing has been performed then the label has also been appended either “1” or “2”.
(PDF)

Figure S8 ***Staphylococcus aureus*** **phylogeny inferred with snpTree (distant reference).** Colors have been omitted from this figure. The sequencing platforms applied are appended to the end of each label. If repetitive sequencing has been performed then the label has also been appended either “1” or “2”.
(PDF)

Figure S9 ***Staphylococcus aureus*** **phylogeny inferred with the novel SNP procedure (distant reference).** Labels are colored according to isolate. The sequencing platforms applied are appended to the end of each label. If repetitive sequencing has been performed then the label has also been appended either “1” or “2”.
(PDF)

Figure S10 ***Staphylococcus aureus*** **phylogeny inferred with the Nucleotide Difference method (distant reference).** Labels are colored according to isolate. The sequencing platforms applied are appended to the end of each label. If repetitive sequencing has been performed then the label has also been appended either “1” or “2”.
(PDF)

Table S1 **Dataset overview.**
(XLSX)
