# Supplementary figures and images for: Solving the Problem of Comparing Whole Bacterial Genomes across Different Sequencing Platforms
Source: PLoS One. 2014 Aug 11;9(8):e104984. doi: 10.1371/journal.pone.0104984 (PMC4128722; doi:10.1371/journal.pone.0104984)

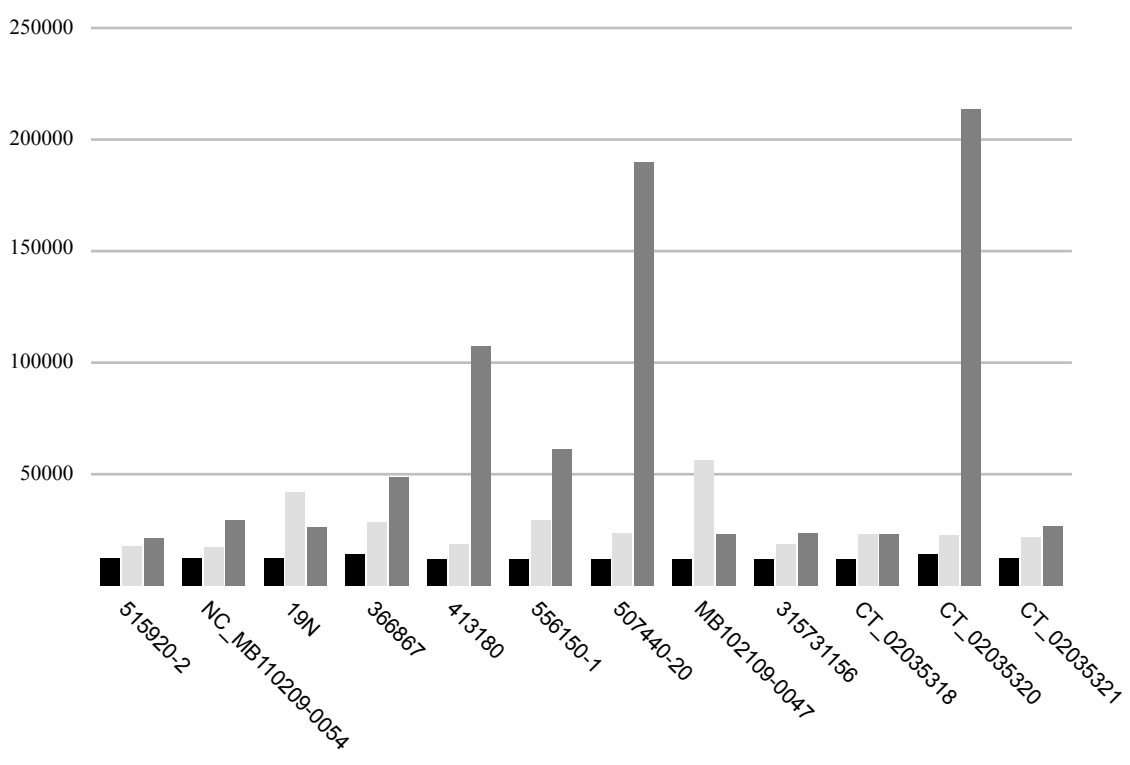

Supplement: Figure S1 — Ignored genome positions in novel SNP procedure ( Salmonella Montevideo dataset). Each cluster of three columns represents the amount of genome locations that are ignored due to the addition of the specific data. Black represents MiSeq data, grey represents Ion Torrent data, and light grey represents 454 data. (PDF) [file pone.0104984.s001.pdf]

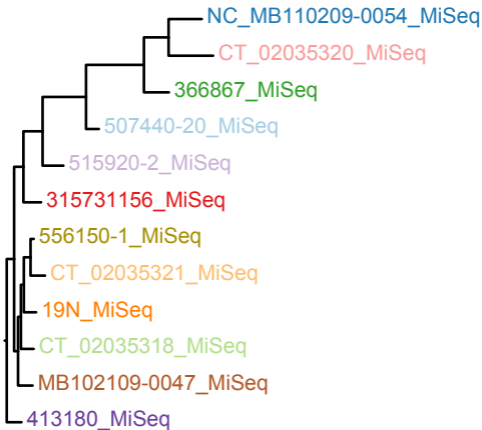

0.2

Supplement: Figure S2 — Salmonella Montevideo phylogeny inferred by snpTree (MiSeq data only). The colors of the labels in the figure correspond to the colors used in the main figures. (PDF) [file pone.0104984.s002.pdf]

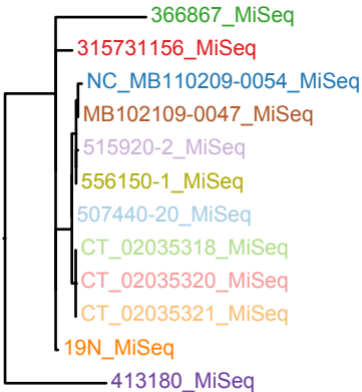

0.2

Supplement: Figure S3 — Salmonella Montevideo phylogeny inferred by the novel SNP procedure (MiSeq data only). The colors of the labels in the figure correspond to the colors used in the main figures. (PDF) [file pone.0104984.s003.pdf]

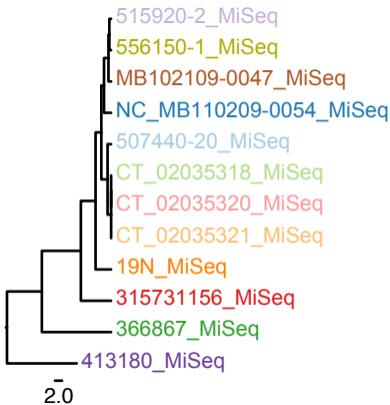

Supplement: Figure S4 — Salmonella Montevideo phylogeny inferred by the Nucleotide Difference method (MiSeq data only). The colors of the labels in the figure correspond to the colors used in the main figures. (PDF) [file pone.0104984.s004.pdf]

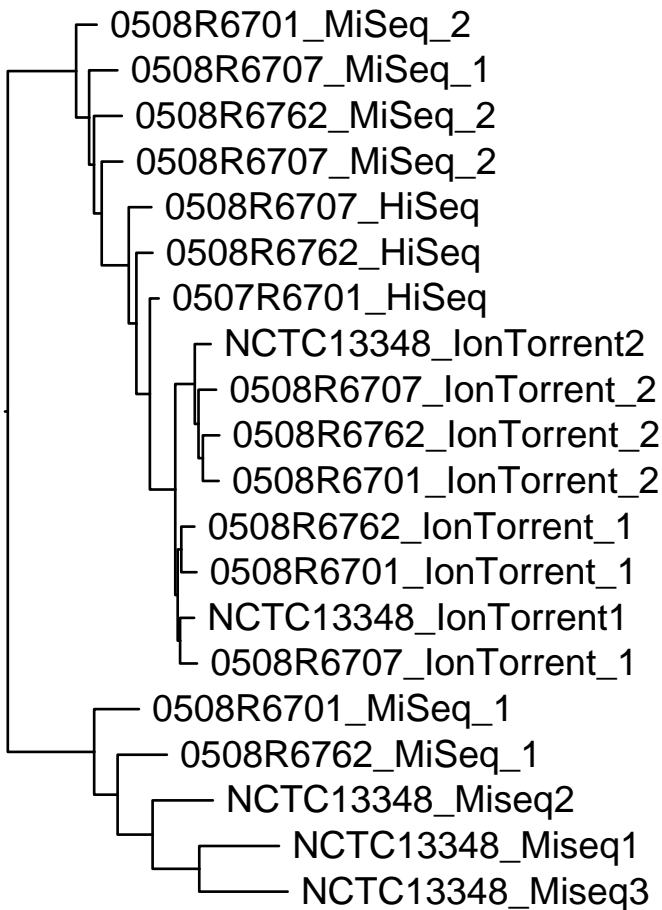

—  
0.08

Supplement: Figure S5 — Salmonella DT104 phylogeny inferred with snpTree (distant reference). Colors have been omitted from this figure. The sequencing platforms applied are appended to the end of each label. If repetitive sequencing has been performed then the label has also been appended either “1” or “2”. (PDF) [file pone.0104984.s005.pdf]

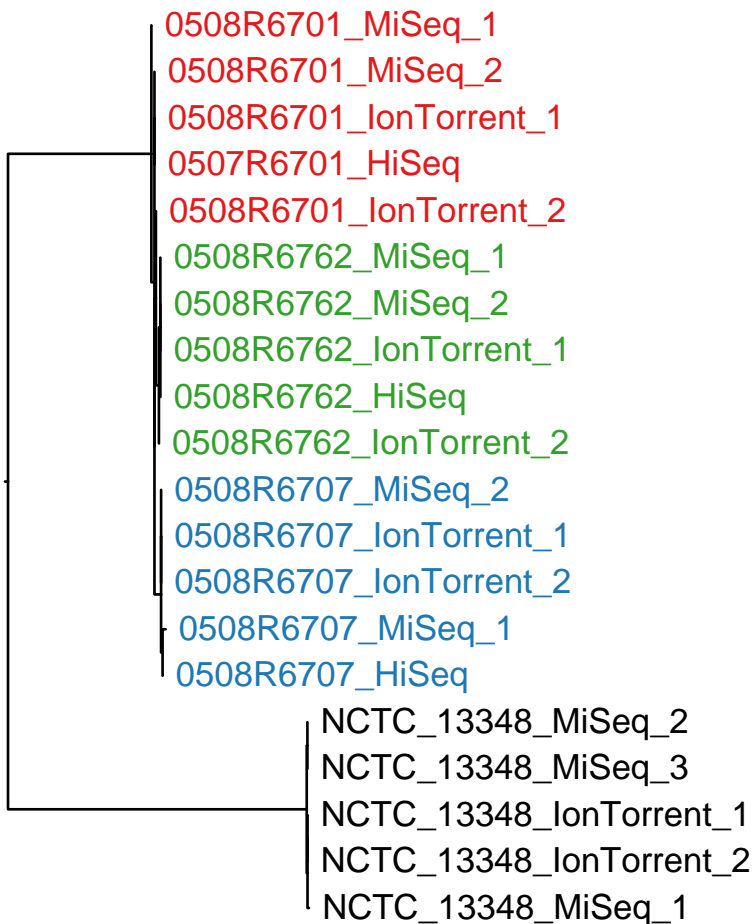

—  
0.5

Supplement: Figure S6 — Salmonella DT104 phylogeny inferred with the novel SNP procedure (distant reference). Labels are colored according to isolate. The sequencing platforms applied are appended to the end of each label. If repetitive sequencing has been performed then the label has also been appended either “1” or “2”. (PDF) [file pone.0104984.s006.pdf]

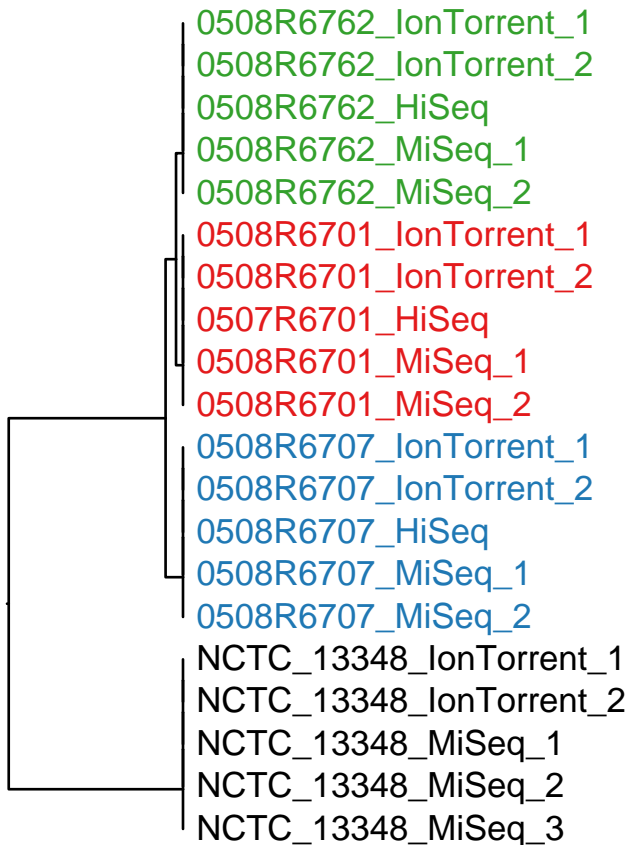

Supplement: Figure S7 — Salmonella DT104 phylogeny inferred with the Nucleotide Difference method (distant reference). Labels are colored according to isolate. The sequencing platforms applied are appended to the end of each label. If repetitive sequencing has been performed then the label has also been appended either “1” or “2”. (PDF) [file pone.0104984.s007.pdf]

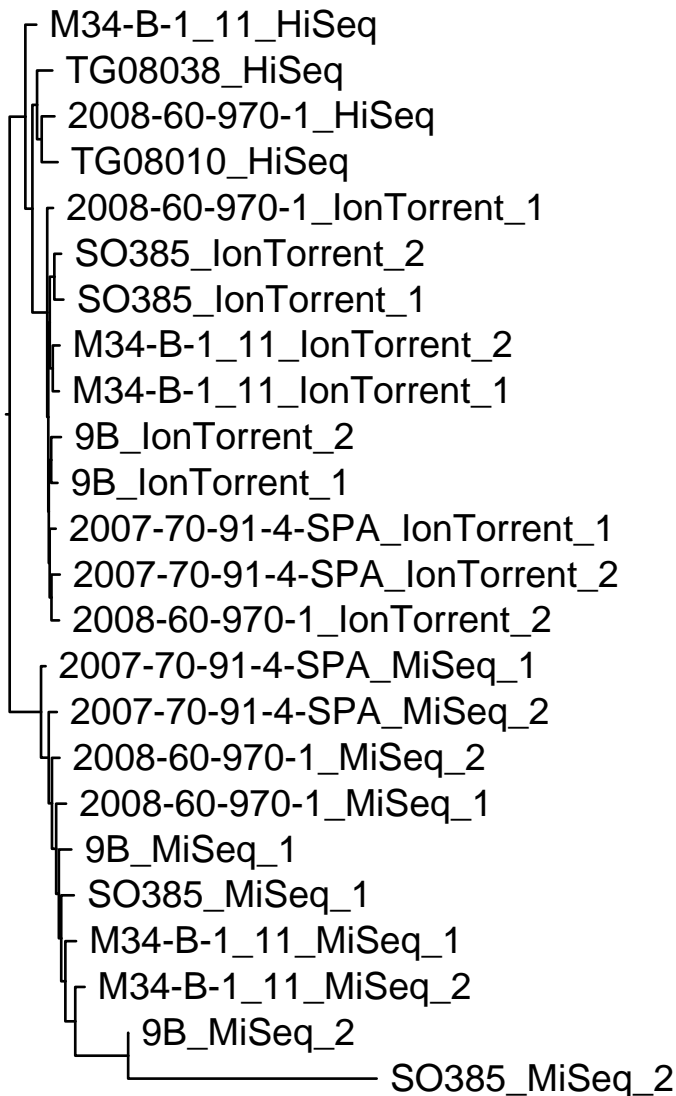

0.2

Supplement: Figure S8 — Staphylococcus aureus phylogeny inferred with snpTree (distant reference). Colors have been omitted from this figure. The sequencing platforms applied are appended to the end of each label. If repetitive sequencing has been performed then the label has also been appended either “1” or “2”. (PDF) [file pone.0104984.s008.pdf]

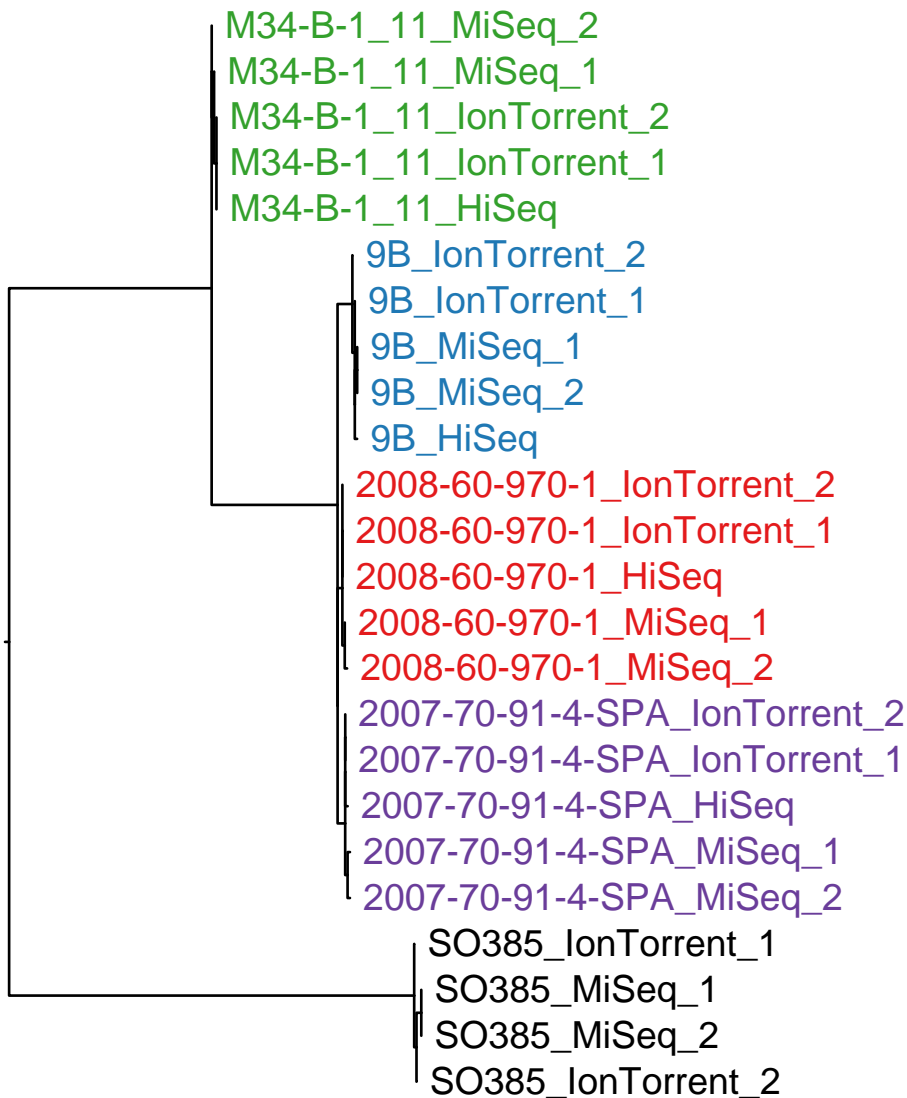

Supplement: Figure S9 — Staphylococcus aureus phylogeny inferred with the novel SNP procedure (distant reference). Labels are colored according to isolate. The sequencing platforms applied are appended to the end of each label. If repetitive sequencing has been performed then the label has also been appended either “1” or “2”. (PDF) [file pone.0104984.s009.pdf]

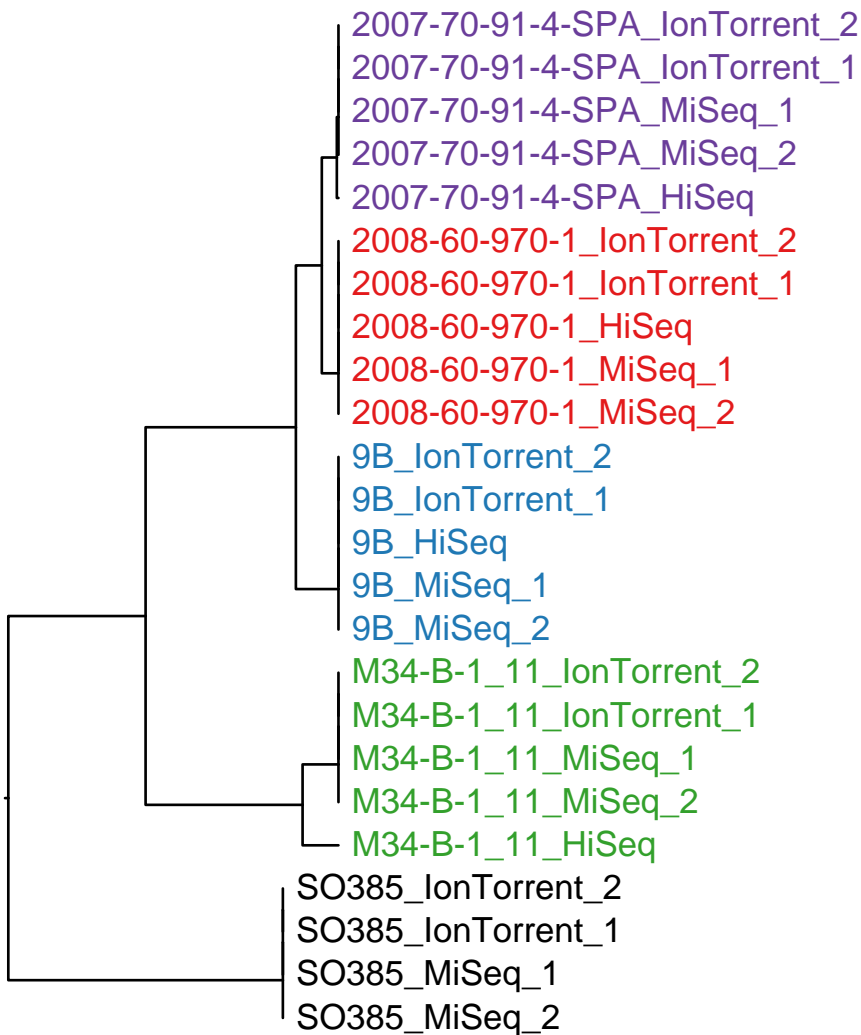

—  
9.0

Supplement: Figure S10 — Staphylococcus aureus phylogeny inferred with the Nucleotide Difference method (distant reference). Labels are colored according to isolate. The sequencing platforms applied are appended to the end of each label. If repetitive sequencing has been performed then the label has also been appended either “1” or “2”. (PDF) [file pone.0104984.s010.pdf]
